# Antibody-Antigen-Adjuvant Conjugates Enable Co-Delivery of Antigen and Adjuvant to Dendritic Cells in Cis but Only Have Partial Targeting Specificity

**DOI:** 10.1371/journal.pone.0040208

**Published:** 2012-07-10

**Authors:** Martin Kreutz, Benoit Giquel, Qin Hu, Ram Abuknesha, Satoshi Uematsu, Shizuo Akira, Frank O. Nestle, Sandra S. Diebold

**Affiliations:** 1 Peter Gorer Department of Immunobiology, King’s College London, Guy’s Hospital, London, United Kingdom; 2 Department of Tumor Immunology, Nijmegen Centre for Molecular Life Sciences, Radboud University Nijmegen Medical Centre, Nijmegen, The Netherlands; 3 Pharmaceutical Science Division, King’s College London, London, United Kingdom; 4 Laboratory of Host Defense, WPI Immunology Frontier Research Center, Osaka University, Osaka, Japan; 5 Cutaneous Medicine and Immunotherapy Unit, King’s College London, Guy’s Hospital, London, United Kingdom; Oklahoma Medical Research Foundation, United States of America

## Abstract

Antibody-antigen conjugates, which promote antigen-presentation by dendritic cells (DC) by means of targeted delivery of antigen to particular DC subsets, represent a powerful vaccination approach. To ensure immunity rather than tolerance induction the co-administration of a suitable adjuvant is paramount. However, co-administration of unlinked adjuvant cannot ensure that all cells targeted by the antibody conjugates are appropriately activated. Furthermore, antigen-presenting cells (APC) that do not present the desired antigen are equally strongly activated and could prime undesired responses against self-antigens. We, therefore, were interested in exploring targeted co-delivery of antigen and adjuvant in cis in form of antibody-antigen-adjuvant conjugates for the induction of anti-tumour immunity. In this study, we report on the assembly and characterization of conjugates consisting of DEC205-specific antibody, the model antigen ovalbumin (OVA) and CpG oligodeoxynucleotides (ODN). We show that such conjugates are more potent at inducing cytotoxic T lymphocyte (CTL) responses than control conjugates mixed with soluble CpG. However, our study also reveals that the nucleic acid moiety of such antibody-antigen-adjuvant conjugates alters their binding and uptake and allows delivery of the antigen and the adjuvant to cells partially independently of DEC205. Nevertheless, antibody-antigen-adjuvant conjugates are superior to antibody-free antigen-adjuvant conjugates in priming CTL responses and efficiently induce anti-tumour immunity in the murine B16 pseudo-metastasis model. A better understanding of the role of the antibody moiety is required to inform future conjugate vaccination strategies for efficient induction of anti-tumour responses.

## Introduction

Targeted delivery of antigen to DC is a very efficient strategy for induction of antigen-specific T cell responses [1]. A number of C-type lectin receptors (CLR) have been explored as target receptors for antibody-mediated antigen delivery, including DEC205 (CD205), CD11c, Dectin-1 and -2, DNGR1 (Clec9A) and DCIR2 [2], [3], [4], [5], [6], [7], [8], [9], [10], [11]. Targeted delivery of antigens to CLR leads to efficient induction of humoral and cellular responses and has been shown to be efficient in inducing anti-viral and anti-tumour immunity [8], [12], [13], [14], [15], [16], [17]. However, while antibody-mediated delivery of antigens to particular APC ensures efficient antigen presentation, the presence of suitable adjuvants is required to guarantee the appropriate activation and, consequently, T cell stimulatory properties of the APC [18]. In the context of anti-tumour immunity induction, synthetic mimics of viral pathogen-associated molecular patterns (PAMP) are of particular interest due to their ability to induce high levels of type I interferon (IFN-I) and as a result of this to promote the initiation of CTL responses [19], [20].

So far, antibody-antigen conjugates have been employed in combination with soluble adjuvants allowing for the activation of APC populations that do not present the delivered antigen. This could potentially lead to counterproductive side effects such as the induction of autoimmune responses. Furthermore, TLR agonists given systemically have been shown to recruit T cell populations to the tissue depleting them from the circulating pool prior to activation [21]. Other adverse effects that were observed upon repeated administration of TLR7 and TLR9 agonists are alterations in the structure of lymphoid follicles and splenomegaly [22], [23]. Strategies that reduce the likelihood of such adverse effects would be beneficial in an immunotherapeutic context. We, therefore, were interested in exploring antibody-mediated co-delivery of antigen and adjuvant to cross-priming DC in cis in form of antibody-antigen-adjuvant conjugates. The co-delivery of antigen and adjuvant would not only allow a reduction in the adjuvant dose making unwanted side effects less likely, but also would ensure that only APC that have taken up the delivered antigen become activated. Furthermore, it has been shown that co-delivery of antigen and adjuvant in form of antigen-adjuvant conjugates or coated beads promotes antigen presentation [24], [25].

To investigate whether antibody-antigen-adjuvant conjugates are efficient in inducing CTL responses and anti-tumour immunity, we generated such conjugates by biochemical cross-linking of the TLR9 agonist CpG 1668 and the class I-restricted peptide epitope of the model antigen OVA to DEC205-specific antibody for a proof-of-principle study. Among the endosomal TLR sensing viral nucleic acids, we chose to trigger TLR9 rather than TLR3 or TLR7/8 for this study for technical and conceptual reasons. CpG 1668 ODN represents a relatively small molecule of defined size, unlike the TLR3 agonist polyI:C, and can be synthesized in a modified form allowing for cross-linking via the introduced sulfhydryl group. More importantly, the mouse CD8α^+^ DC subset specialized in cross-priming and primarily targeted by DEC205 antibody expresses TLR9 and TLR3, but not TLR7 and mouse TLR8 is thought to be non-functional [26], [27], [28]. In an alternative approach, we generated equivalent conjugates by biochemical conjugation of the TLR9 agonist to antigen fusion antibody containing a heavy chain genetically fused to the full-length antigen. We characterized these conjugates with regard to their efficacy in inducing CTL response and anti-tumour immunity and investigated their uptake by DC.

## Results

### Generation of Antibody-antigen-adjuvant Conjugates

Antibody-antigen-adjuvant conjugates for targeted co-delivery of antigen and adjuvant to DEC205-expressing cells were generated using the cross-linker sulfo-SMCC for conjugation of a DEC205-specific antibody with the MHC-class I restricted peptide epitope SIINFEKL of the model antigen OVA and the TLR9 agonist CpG 1668 (DEC-OVA-CpG). Conjugates were characterised upon SDS gel electrophoresis by sequential staining of the gel with ethidium bromide and Comassie blue. As expected, the size of the antibody-antigen-adjuvant conjugates was greater than unconjugated antibody and consisted of a nucleic acid moiety in addition to the protein as detected by ethidium bromide and Comassie Blue staining, respectively (Figure S1A,B). The ethidium bromide staining also confirmed that unconjugated CpG had been successfully removed from the purified conjugate preparation (Figure S1C).

The composition of the conjugates was determined by photometric analysis of the nucleic acid content in combination with the quantification of the biotin-labelled OVA peptide moiety and the analysis of the protein concentration. An overview over the molar composition of the different antibody-antigen-adjuvant conjugates is given in Table 1. Overall, on average three CpG ODN were conjugated to each antibody molecule, while the ratio between antibody and peptide antigen was ranging between 1∶1 to 1∶3 for different DEC-OVA-CpG conjugate preparations.

**Table 1:** Molar composition of generated conjugates.

Conjugate	Molar composition		
	Antibody [mol]	Antigen [mol]	Adjuvant [mol]
DEC-OVA-CpG	1	2.2±1.1	3.9±1.1
DEC-OVA-GpC	1	3.2±1.7	2.8±1.3
DEC-OVA	1	2.8±1.1	N/A
iso1-OVA-CpG	1	1.6±0.4	3.4±1.1
iso2-OVA-CpG*	1	1.3	4.3
iso-OVA*	1	1.7	N/A
DEC-Fus-CpG∇	1	2	5.3±0.18
DEC-Fus-CpG	1	2	10.4±1.3
iso-Fus-CpG	1	2	10.5±0.4
OVA-CpG∇	N/A	1	2.0±0.21
OVA-CpG	N/A	1	1.1±0.14

The table depicts the composition of the generated conjugates regarding the molar ratios of the antibody, the antigen (either SIINFEKL peptide or OVA protein) and the nucleic acid adjuvant (CpG or GpC ODN) moieties. Where several batches of conjugates were generated, mean values +/− SD are displayed.

* Only one batch of these conjugates was generated.

N/A - not applicable.

∇ These preparations of DEC-Fus-CpG and OVA-CpG were used for direct comparison of these conjugates allowing for the application of similar amount of adjuvant when normalizing for the antigen dose.

### DEC-OVA-CpG Conjugate Induces TLR9-dependent CTL Priming

**Figure 1:**
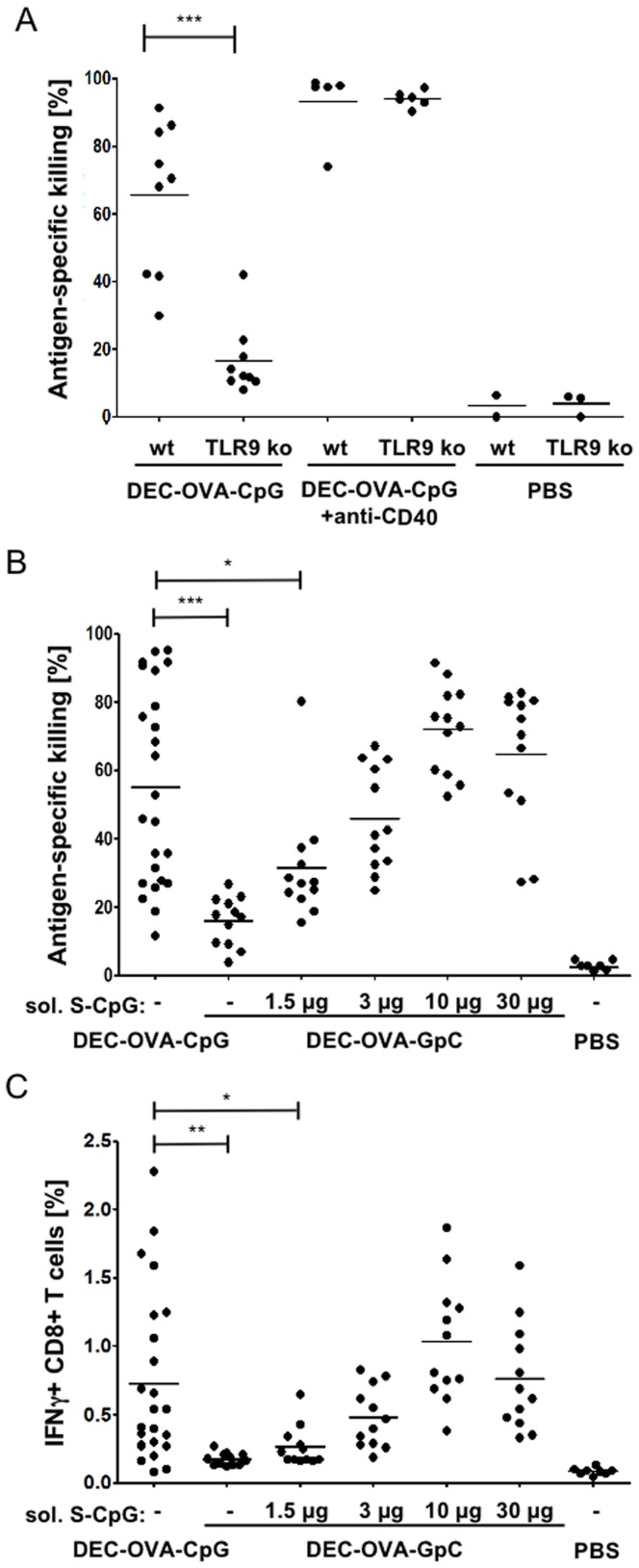
CTL priming by DEC-OVA-CpG conjugate is TLR9-dependent and more efficient than co-injection of control conjugate and soluble CpG. (A) C57BL/6 and TLR9^−/−^ mice were immunised into the footpad with 6–8 µg of DEC-OVA-CpG conjugate with or without 25 µg anti-CD40 antibody or received PBS injections. At day 5 after immunisation, target cells were injected intravenously and the following day, antigen-specific killing was analysed by flow cytometric analysis of splenocyte preparations. The depicted data are pooled from three independent experiments using three different conjugate preparations. (B) C57BL/6 mice were immunised into the footpad with 8 µg of DEC-OVA-CpG or DEC-OVA-GpC control conjugate. The control conjugate was administrated alone or in combination with increasing doses of soluble S-CpG as indicated. 1.52 µg co-injected soluble S-CpG equals the amount of S-CpG delivered in conjugated form with 8 µg of DEC-OVA-CpG. Control mice were injected with PBS. At day 5 after immunisation, target cells were injected and the in vivo CTL assay was analysed the following day as described above. (C) Additionally, splenocytes were cultured over night in the presence of 1 µM SIINFEKL peptide and the frequency of IFNγ-producing CD8^+^ T cells was determined the next day after 3 hours of incubation in the presence of Brefeldin A. Data are pooled from four independent experiments (A-C). Each dot represents the CTL response or the frequency of IFNγ-producing CD8^+^ T cells of an individual mouse, while the average values for each group are depicted as a bar.

To test the ability of the DEC-OVA-CpG conjugate to induce an antigen-specific immune response, mice were vaccinated by footpad injection and in vivo CTL assays were performed six days later. Vaccination with DEC-OVA-CpG conjugate induced a CTL response in C57BL/6 mice with an average of 65.5%±22.2 of peptide-pulsed target cells being killed (Figure 1A). Antigen-specific target cell killing was largely reduced in TLR9 ko mice indicating that CTL priming is dependent on innate immune activation by the conjugate. Mice vaccinated with conjugate in the presence of anti-CD40 antibody as alternative TLR-independent adjuvant mounted a comparable CTL response independently of TLR9 sufficiency.

### DEC-OVA-CpG is More Efficient in Inducing CTL Priming than Control Conjugate Co-injected with Soluble CpG

We were interested in evaluating the efficiency by which DEC-OVA-CpG conjugate induces a CTL response in comparison to an equivalent dose of a control conjugate co-injected with soluble CpG ODN. To control for the size and the biochemical make-up of the conjugates, we compared DEC-OVA-CpG to DEC-OVA-GpC conjugate, the latter containing an ODN that does not induce innate immune activation at the used dose [29]. The non-stimulatory DEC-OVA-GpC control conjugate was co-injected with increasing doses of soluble CpG ODN with 1.5 µg being equivalent to the dose of CpG administered in conjugated form with DEC-OVA-CpG. Interestingly, co-administration of increasing doses of soluble CpG ODN resulted in a bell-shaped dose response with 30 µg of co-injected CpG leading to less antigen-specific killing of target cells than co-injection of 10 µg of the TLR9 agonist (Figure 1B). High doses of CpG have been shown to induce higher levels of the anti-inflammatory cytokine interleukin-10, which could lead to suboptimal CTL priming [30]. More importantly, vaccination with DEC-OVA-CpG induced a significantly higher average CTL response (55.0%±28.6) than the DEC-OVA-GpC control conjugate injected with the equivalent amount of 1.5 µg soluble CpG ODN (31.6%±16.8).

The frequency of interferon-γ (IFNγ)-producing CD8 T cells as monitored by intracellular cytokine staining of ex vivo restimulated splenocytes from vaccinated mice, resulted in the same pattern as observed for the in vivo CTL assay confirming that DEC-OVA-GpC conjugate co-injected with an equivalent amount of soluble CpG is less efficient in inducing a CD8 T cell response than DEC-OVA-CpG conjugate (Figure 1C). Thus, despite the high variability in CTL induction in mice vaccinated with DEC-OVA-CpG, our data indicate that CpG co-delivered with the antigen in cis in form of antibody-antigen-adjuvant conjugate is more efficient in promoting CTL priming than antibody-antigen conjugate co-administered with soluble CpG in trans.

### Induction of Antigen-specific Killing by DEC-OVA-CpG Conjugate is not Dependent on DEC205-mediated Targeting

**Figure 2:**
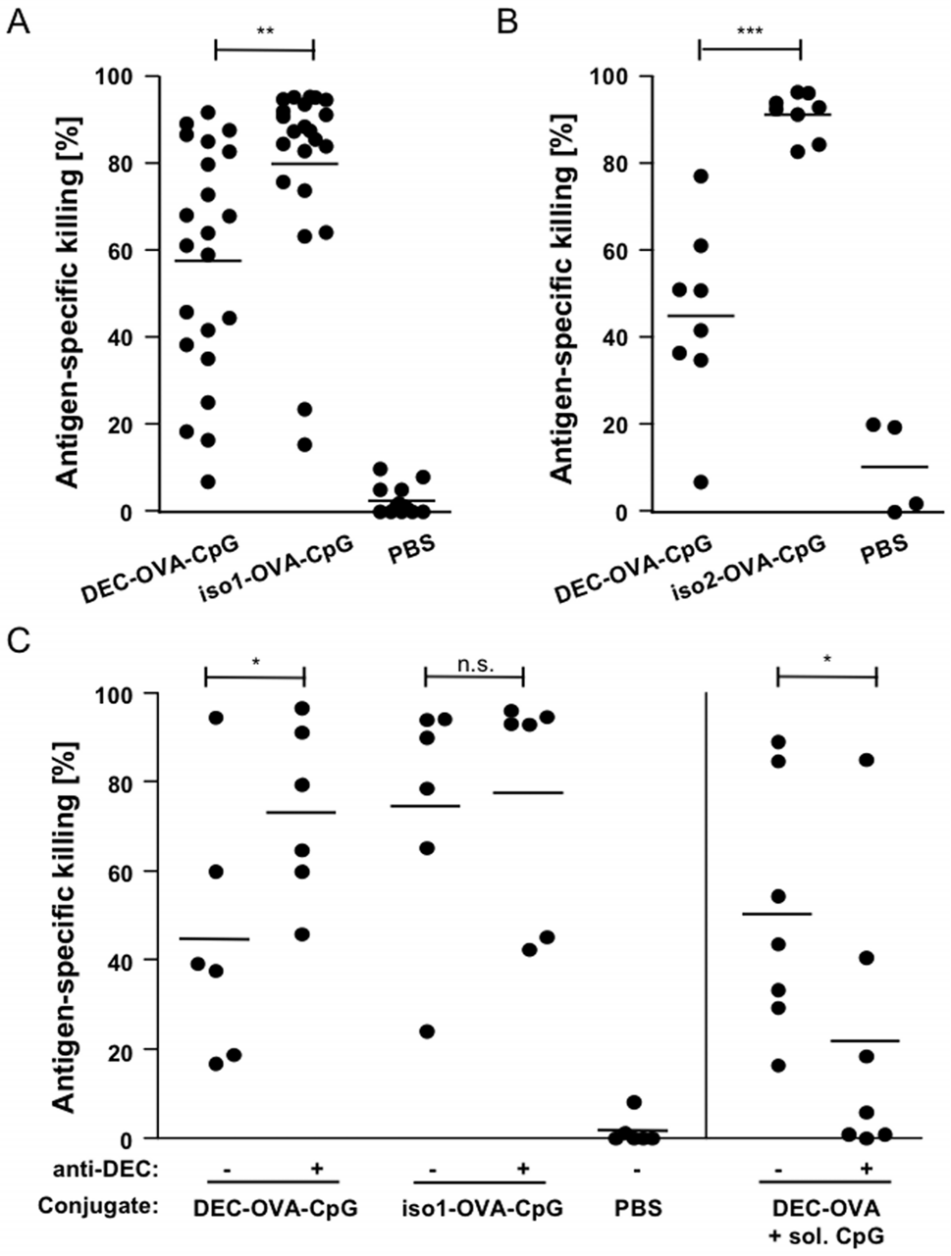
DEC205-independent CTL induction by antibody-antigen-adjuvant conjugates. (A–C) C57BL/6 mice were immunised into the footpad with 8 µg of DEC-OVA-CpG, iso-OVA-CpG or DEC-OVA conjugate. In (C) immunisations took place in the presence or absence of 200 µg of anti-DEC205 antibody and DEC-OVA was co-injected with 10 µg soluble CpG ODN. Control mice received PBS injections. At day 5 or 6 after immunisation, target cells were injected intravenously. The following day, the in vivo CTL assay was analysed by flow cytometry. Data in (A) are pooled from 7 experiments while the graphs in (B and C) contain data from 2 independent experiments. In (C) a one-tailed Student’s t-test was used for statistical analysis.

Antibody-antigen conjugates targeting DEC205 on antigen-presenting DC confer immunity in a DEC205-dependent manner [3]. Consequently, antibody-antigen conjugates generated with isotype control antibody are inefficient in inducing an antigen-specific immune response [2], [3]. To investigate the requirement for DEC205-targeted delivery of the antibody-antigen-adjuvant conjugates, we compared the induction of CTL responses in DEC-OVA-CpG vaccinated mice to those in mice immunised with equivalent conjugates generated with an isotype control antibody (iso1-OVA-CpG). Surprisingly, vaccination with iso1-OVA-CpG resulted in excellent antigen-specific killing of target cells, even exceeding the response induced by the DEC-OVA-CpG conjugate (Figure 2A). To confirm this unexpected result, we tested an additional control conjugate generated with a different isotype control antibody (iso2-OVA-CpG; Figure 2B). Also in this case, the CTL response was stronger for the isotype control antibody-containing conjugate than for the DEC205-targeted antibody-antigen-adjuvant conjugate. Endotoxin levels of all conjugates were below 10 U/mg suggesting that endotoxin contamination was not responsible for the increased CTL induction observed with the isotype control antibody-containing conjugates. The results, therefore, suggest that targeting of antibody-antigen-adjuvant conjugates to DEC205 is reducing rather than enhancing the immunogenicity of the tested antibody-antigen-adjuvant conjugates. This effect is, however, not observed for the corresponding antibody-antigen conjugates co-injected with soluble TLR9 agonist (Figure S2) implicating that the addition of a nucleic acid moiety alters the functional properties of the conjugates.

To further investigate this, we blocked DEC205-mediated conjugate delivery by co-injecting a high dose of unconjugated anti-DEC205 antibody together with DEC-OVA-CpG conjugate. Unconjugated DEC205 antibody decreased antigen-specific target cell killing in control mice that were vaccinated with DEC-OVA conjugate in the presence of soluble CpG ODN indicating that the competition for DEC205 reduced DEC205-specific delivery of this conjugate (Figure 2C). In contrast, in mice vaccinated with DEC-OVA-CpG conjugate, CTL-mediated killing was improved when DEC205-mediated delivery of the conjugate was impaired by the presence of unconjugated DEC205-specific antibody and reached levels observed for iso-OVA-CpG conjugate (Figure 2C). As expected, CTL induction by iso-OVA-CpG conjugates was unaffected by the presence of DEC205-specific antibody (Figure 2C). These blocking experiments support the conclusion that DEC205-mediated targeting of the tested conjugates reduces rather than enhances their immunogenicity.

### The SIINFEKL Peptide and the Nucleic Acid Moieties Influence the Binding of Antibody-antigen-adjuvant Conjugates

To investigate how the addition of a nucleic acid moiety influences the binding of the conjugates, we stained CD11c-enriched splenocytes with antibody-antigen and antibody-antigen-adjuvant conjugates or unconjugated antibody, which were either directly fluorescently labelled or indirectly detected with secondary reagents as indicated. Direct and indirect detection of conjugates produced the same staining pattern ruling out the possibility that a contamination with fluoescently labelled antibody not conjugated to antigen or CpG ODN, or that the addition of an additional moiety in form of the fluorescent dye skewed the binding profile of the conjugates (Figure S3). In addition, dose titrations for the different conjugates were performed to exclude the possibilty that saturating doses mask differences in the strength of binding (Figure S3). Interestingly, staining with DEC205-specific antibody was saturated at doses from 10 nM upwards, whereas saturation of binding of OVA peptide-containing conjugates was observed for doses from 90 nM upwards (Figure S3). This suggests that DEC205-specific antibody-containing conjugates may lose some of their binding capacity due to the conjugation process. In all subsequent staining experiments a dose of 30 nM conjugate was used to exclude saturation of DEC205-mediated binding on CD8α^+^ DC.

**Figure 3:**
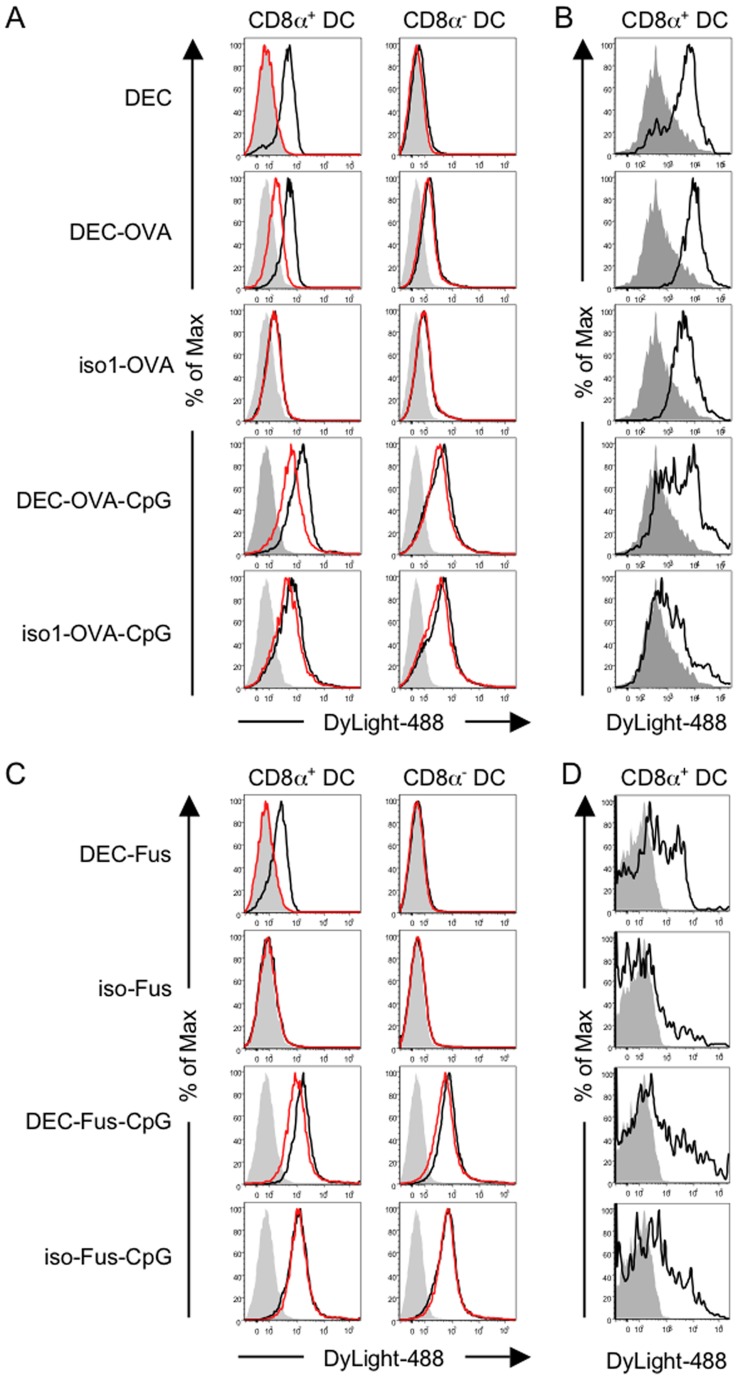
Addition of a CpG ODN moiety alters binding and in vivo targeting of antibody-antigen conjugates. (A, C) CD11c-enriched splenocytes from C57BL/6 were stained with 30 nM DyLight488-labelled antibody or conjugate in combination with CD11c- and CD8α-specific antibodies. For blocking of DEC205-mediated conjugate binding, unlabelled DEC205-specific antibody (100 µg/ml) was added to the staining mix. Binding of antibody or conjugate was analysed by flow cytometry gating on CD8α^+^ versus CD8α^−^ DC. The overlays show staining with DEC205-specific antibody (DEC) or the different conjugates in the absence (black histograms) or in the presence of blocking DEC205 antibody (red histograms) as indicated. Staining with isotype control antibody is shown as solid grey histograms. (B, D) DyLight488-labelled antibodies and conjugates were injected into the footpad of C57BL/6 mice. Single cell suspensions from popliteal lymph nodes were prepared 6 hours later and splenocytes suspension were analysed directly (B) or after enrichment of MHC class II^+^ cells by magnetic cell sorting (D). Cells were stained with CD11c- and CD8α-specific antibodies and the percentage of DyLight488^+^ CD8α^+^ DC was determined by flow cytometry. Background staining of CD8α^+^ DC from mice injected with labelled isotype control antibody (B) or PBS (D) is shown as solid grey histograms overlayed with DyLight488-staining of CD8α^+^ DC from mice that received labelled DEC205-specific antibody or conjugates as indicated (black histograms). Data are representative of at least 2 independent experiments.

As expected, DEC205-specific antibody predominantly bound to splenic CD8α^+^ DC confirming that these cross-priming DC express high levels of DEC205 (Figure 3A). In contrast, DEC-OVA conjugate stained CD8α^+^ and CD8α^−^ DC subsets as did iso-OVA conjugate (Figure 3A). However, DEC-OVA conjugate bound more efficiently to CD8α^+^ DC than to the CD8α^-^ DC subsets, whereas iso-OVA conjugate showed only weak binding to these DC subsets irrespective of DEC205 expression (Figure 3A). The binding of DEC-OVA-CpG conjugate to CD8α^+^ and CD8α^−^ DC was improved as compared to the binding of DEC-OVA conjugate and was almost indistinguishable from binding of the equivalent isotype control conjugate (Figure 3A).

In order to determine the extent of DEC205-mediated binding of the conjugates, we performed blocking experiments with unlabelled DEC205-specific antibody. DEC-OVA and the corresponding isotype control antibody-containing conjugates showed low levels of DEC205-independent binding to CD8α^+^ and CD8α^−^ DC. However, the DEC-OVA conjugate retained the capacity to bind to DEC205 as shown by the partial block in conjugate binding in the presence of excess DEC205 antibody (Figure 3A). To investigate whether the binding of DEC-OVA and iso1-OVA conjugate was mediated by the peptide moiety, we performed additional blocking experiments with SIINFEKL peptide and also tested equivalent conjugates with a longer OVA-specific peptide referred to as OVAlong. Surprisingly, the OVAlong-containing antibody-antigen conjugates did not mediate DEC205-independent binding (Figures S3 and S4) suggesting that peptide-mediated binding of DEC-OVA and iso1-OVA conjugate is a SIINFEKL-specific artefact. Furthermore, blocking with unconjugated SIINFEKL peptide led to a partial reduction in binding of DEC-OVA and to a complete reduction in binding of iso1-OVA conjugate by CD8α^−^ DC supporting the notion that SIINFEKL peptide mediates binding of these antibody-antigen conjugates to DC (Figure S4A). Blocking of DEC205- and SIINFEKL-mediated binding of DEC-OVA conjugate, both only led to a partial reduction in conjugate binding by CD8α^+^ DC supporting the conclusion that both conjugate moieties mediate binding independently (Figures 3A and S4A). In contrast, binding of DEC-OVAlong was completely blocked in the presence of blocking DEC205 antibody (Figure S4A).

For conjugates containing a CpG moiety, blocking experiments with DEC205 antibody or SIINFEKL peptide were ineffective or only slightly reduced conjugate binding (Figure S4A). For further characterisation of the binding modalities of our conjugates, we also performed blocking experiments with polydT17-G12 ODN in order to inhibit CpG-mediated binding. Conjugates without the CpG moiety showed no reduction in binding in the presence of polydT17-G12 in contrast to antibody-antigen-adjuvant conjugates (Figure S4A). We chose polydT17-G12 ODN for blocking of CpG-mediated binding, since it lacks immunostimulatory activity at high concentrations unlike GpC ODN. Interestingly, polydT17-G12 partially inhibited binding of DEC-OVA-CpG on CD8α^−^ but not on CD8α^+^ DC probably due to the presence of DEC205-mediated binding by the latter DC subset (Figure S4A). However, the reduction in binding of CpG-containing DEC-OVA-CpG and iso1-OVA-CpG conjugates by CD8α^−^ DC was not absolute, most likely due to the presence of SIINFEKL-mediated binding.

While these binding studies suggest that the addition of the SIINFEKL peptide moiety, as well as the addition of a nucleic acid moiety influence the binding of the conjugates, it is unclear how this affects uptake in vivo. To investigate this, we injected fluorescently labelled antibodies, antibody-antigen and antibody-antigen-adjuvant conjugates into C57BL/6 mice by footpad injection and analysed uptake of the fluorescent marker by CD11c^+^ cells in the draining lymph node. Unfortunately, we could not discriminate between the different lymph node DC populations, since the injection of DEC205-specific antibody leads uniformly to the down-regulation of DEC205 on the otherwise DEC205^+^ DC subsets. While DEC205-specific antibody efficiently stains CD8α^+^ lymph node DC, isotype control antibody did not as efficiently associate with this DC population (Figure 3B). Similar to what had been observed in vitro, antibody-antigen conjugates were efficiently taken up by CD8α^+^ DC and CD8α^−^ DC in vivo irrespective of the antibody specificity (Figure 3B). Interestingly, levels of uptake of DEC-OVA-CpG conjugate by a subpopulation of CD8α^+^ DC was equivalent to the uptake of DEC205-specific antibody and DEC-OVA conjugate, but the other subpopulation of CD8α^+^ DC stained only poorly for the fluoresecent dye. However, a clear difference in uptake between DEC-OVA-CpG and iso1-OVA-CpG conjugates was observed suggesting that upon addition of a CpG moiety the conjugates regain some level of targeting specificity. These data confirm that the conjugation of SIINFEKL peptide and CpG ODN to antibody for the generation of antibody-antigen-adjuvant conjugates alters the binding pattern and the uptake of these conjugates. Similar results were obtained for antibody-antigen-adjuvant conjugates generated with non-stimulatory GpC ODN (data not shown). However, the difference in uptake between antibody-antigen-adjuvant conjugates containing DEC205-specific versus isotype control antibody does not correlate with their efficiency in CTL induction.

The finding that the addition of the SIINFEKL peptide alters the binding specificity of the conjugates was particularly surprising. To exlude SIINFEKL-mediated binding of conjugates, we performed similar experiments using antigen fusion antibody containing full-length OVA fused to the constant region of the antibody heavy chain [3]. As expected from our experiments with DEC-OVA and DEC-OVAlong conjugates, genetically engineered DEC-OVA fusion antibody (DEC-Fus) showed a preference for binding to DEC205-high CD8α^+^ DC in vitro and did not bind to the CD8α^−^ DC subsets (Figure 3C). Isotype control antibody-antigen fusion protein failed to bind to any of the DC subsets with great efficiency (Figure 3C). We produced second-generation antibody-antigen-adjuvant conjugates by biochemically linking CpG ODN to antigen fusion antibodies (Figure S5). The CpG-containing fusion antibody conjugates (DEC-Fus-CpG and iso-Fus-CpG) bound equally well to all CD8α^−^ and CD8α^+^ DC irrespective of the antibody specificity (Figure 3C). A dose titration of the conjugates revealed that their capacity to bind CD8α^+^ DC was identical to that of peptide-containing conjugates and saturating doses were reached at 90 nM and above (Figure S3). Due to the absence of peptide-mediated binding, DEC-Fus conjugate binding was entirely blocked by DEC205-specific antibody as expected (Figures 3C and S4B). As seen before for DEC-OVA-CpG and iso1-OVA-CpG conjugates, binding of CpG-containing conjugates is partially mediated by the nucleic acid moiety and is reduced in the presence of polydT17-G12 ODN (Figure S4B). When fluorescently labelled antigen fusion antibodies (DEC-Fus and iso-Fus) were injected into the footpad of mice, DEC205-specific delivery of antigen fusion antibody to CD8α^+^ DC was observed (Figure 3D). In contrast, the antigen fusion antibodies conjugated to CpG ODN (DEC-Fus-CpG and iso-Fus-CpG), bound to CD8α^+^ DC irrespective of the antibody-specificity and were equally well delivered to CD8α^−^ DC (Figure 3D and data not shown).

In summary, the data show that the addition of a CpG moiety to antibody-antigen conjugates alters their binding properties and, thus, may also make the targeted delivery of the antigen partially independent of the antibody component.

### Antibody-antigen-adjuvant Conjugates are More Efficient in Priming CTL than the Equivalent Antigen-adjuvant Conjugates Despite their Partial Antibody-independent Binding

**Figure 4:**
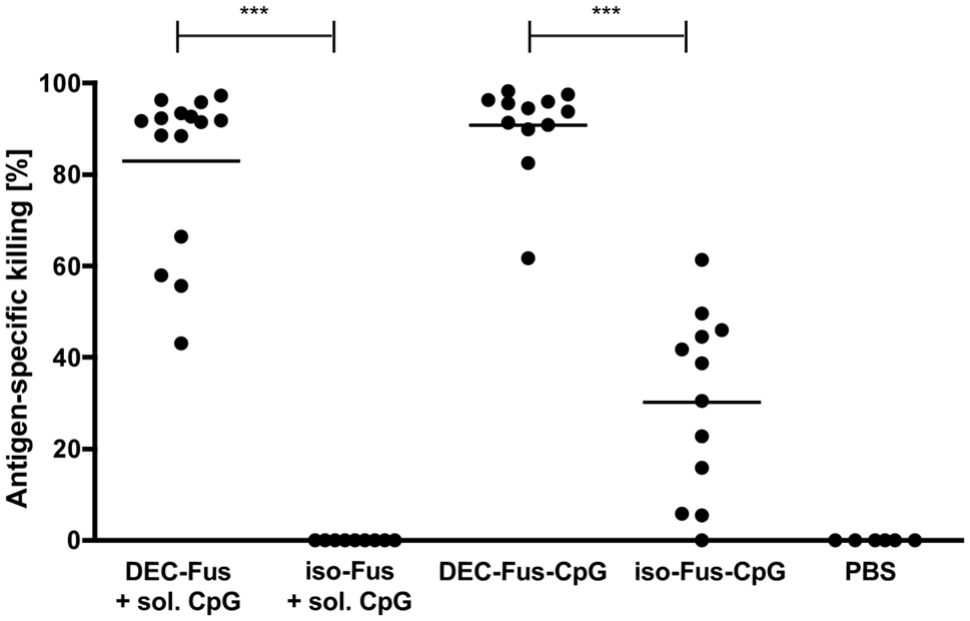
CTL induction by antigen fusion antibody-adjuvant conjugate is promoted by DEC205-mediated targeting. C57BL/6 mice were immunised into the footpad with 8 µg of ovalbumin fusion antibody either containing DEC205-specific (DEC-Fus) or isotype control antibody (iso-Fus) in combination with 10 µg soluble CpG ODN. Alternatively, mice were immunised with 8 µg of CpG-containing conjugates generated with Dec205-specific (DEC-OVA-CpG) or isotype control antigen fusion antibody (iso-Fus-CpG). Control mice received PBS injections. At day 5 after immunisation, in vivo CTL assays were performed by injecting target cells intravenously and by analysing antigen-specific killing the following day. The depicted data are pooled from 3–5 independent experiments with three mice per group for each experiment. Each dot represents the CTL response of an individual mouse. The average antigen-specific killing for each group is depicted as a bar.

To determine the extent to which the fusion antibody conjugates induce antigen-specific CTL-mediated killing, we vaccinated mice and determined antigen-specific target cell killing by in vivo CTL assay. As expected, DEC205-specific antigen fusion antibody co-injected with soluble CpG ODN was efficient in inducing a CTL response in contrast to antigen fusion isotype control antibody (Figure 4). CpG-containing conjugates generated with isotype control antigen fusion antibody induced significantly lower levels of CTL-mediated killing than DEC205-targeted conjugate (Figure 4). This result suggests that exclusive CpG-mediated targeting of antigen fusion antibody-CpG conjugates is less efficient in promoting CTL induction than in combination with DEC205-mediated targeting.

**Figure 5:**
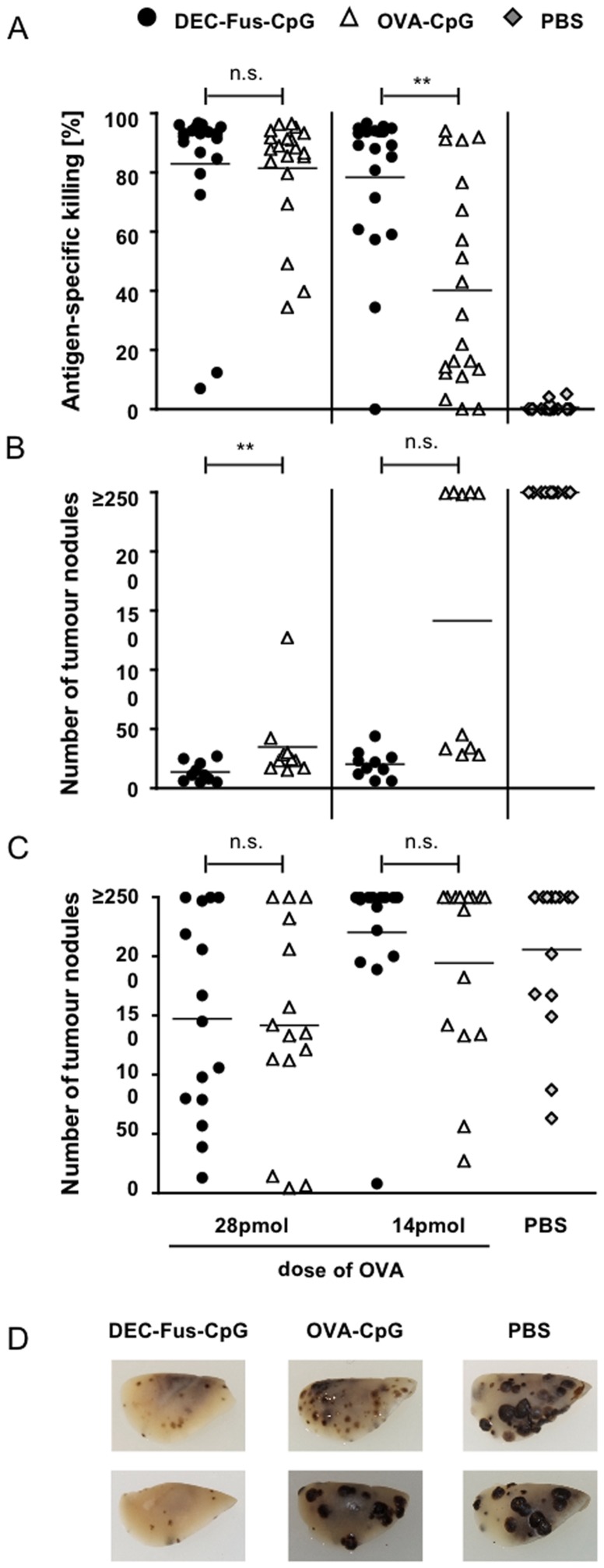
Antigen fusion antibody-adjuvant conjugate is superior in inducing anti-tumour immunity in comparison to antigen-adjuvant conjugate. C57BL/6 mice were immunised into the footpad with comparable doses of antigen (14 pmol and 28 pmol) in form of fusion antibody conjugated to CpG (DEC-Fus-CpG) or antigen-adjuvant conjugate (OVA-CpG). Control mice were injected with PBS. (A) At day 5 after immunisation, in vivo CTL assays were performed by intravenous injection of target cells. Antigen-specific killing of target cells was analysed the following day. The depicted data are pooled from 4 independent experiments using two different conjugate batches for each conjugate type. (B) 30 days post immunisation, OVA-expressing B16 melanoma cells were injected intravenously and 18 days later, the number of lung nodules was determined. Data from two independent experiments are shown. (C) For the therapeutic tumour model, mice were inoculated intravenously with OVA-expressing B16 melanoma cells and 6 days later mice were vaccinated by footpad injection. The number of tumour nodules was evaluated 18 days after tumour cell injection. Data from three independent experiments are shown. Each symbol representing an individual mouse while the average antigen-specific killing (A) or number of lung nodules (B and C) for each group is depicted as a bar. (D) Representative photographs of the fixed left upper lung lobes of mice treated with 28 pmol of conjugates as described in (C) to demonstrate that the number of tumour nodules was not necessarily reduced upon treatment, but that tumour nodules were much smaller than in mock-treated mice especially upon vaccination with DEC-Fus-CpG conjugate.

To further consolidate the hypothesis that antibody-mediated targeting of DEC205 by conjugates containing a CpG moiety contributes to CTL priming, we compared antibody-antigen-adjuvant conjugates with antigen-adjuvant conjugates. To address this question, we generated conjugates of OVA protein and CpG ODN (OVA-CpG) by biochemical linkage with the cross-linker sulfo-SMCC [24], [31], [32]. The calculated molecular weight of the OVA-CpG conjugate is 5 times smaller than the molecular weight of the corresponding antibody-antigen-adjuvant conjugate (DEC-Fus-CpG) and the fusion antibody conjugate contains two mol of antigen per mol of conjugate in contrast to one mol of antigen per mol of antigen-adjuvant conjugate. We injected mice with different, but comparable doses of these conjugates normalising for the amount of antigen applied and evaluated the induced CTL responses. At a dose of 2 µg, DEC-Fus-CpG leads to average antigen-specific killing of 78.3% +/−25.1 in vaccinated mice (Figure 5A). A dose of 0.8 µg OVA-CpG provides the equivalent amount of antigen (14 pmol) and a similar dose of adjuvant compared to 2 µg of the antibody-antigen-adjuvant conjugate, but only leads to 40.17% +/−34.3 of target cell killing (Figure 5A). When a higher equivalent dose (28 pmol of antigen) is used for vaccination, OVA-CpG and DEC-Fus-CpG induce comparable levels of antigen-specific killing (Figure 5A).

Next, we tested these conjugates for the induction of anti-tumour immunity in a prophylactic B16 pseudo-metastasis model. Vaccination with the higher dose of Dec-Fus-CpG and OVA-CpG conjugates delivering 28 pmol of antigen protected the mice from tumour growth. However there was a small but significant difference in the level of protection with the antibody-containing conjugates being more effective in inducing anti-tumour immunity (Figure 5B). However, when vaccinating the mice with the lower dose of antigen, strong inter-experimental variability was observed. Remarkably, vaccination with 0.8 µg of OVA-CpG conjugate (14 pmol of antigen) failed to protect the mice from tumour growth in one of the two experiments, even though this dose led to a measurable CTL response (Figure 5). Interestingly, 1 µg of DEC-Fus-CpG conjugate equalling a dose of 7 pmol of antigen was still sufficient to largely protect the mice from tumour challenge, despite the fact that at this dose CTL activity was highly variable with 44.5% +/−43.6 of antigen-specific killing (Figure S6).

We also evaluated the therapeutic potential of the conjugates in the B16 pseudo-metatstasis model and treated mice 6 days after tumour cell inoculation. At an antigen dose of 28 pmol, mice vaccinated with either conjugate were partially protected from tumour challenge with regard to the number of visible tumour nodules (Figure 5C; p<0.05 for DEC-Fus-CpG and OVA-CpG versus mock, respectively). At the lower antigen dose of 14 pmol, the number of tumour nodules was not significantly reduced compared to untreated mice (Figure 5C). However, the treatment showed clear beneficial influence on the size of the tumour nodules, which were very small in comparison to the nodules observed in untreated mice (Figures 5D and S7). The reduction in the size of the tumour nodules was more prominent in mice vaccinated with Dec-Fus-CpG conjugates as compared to OVA-CpG conjugates. Unfortunately, this observation was not quantifiable.

Taken together, these data imply that the targeted delivery of antigen and adjuvant using a DEC205-specific antigen fusion antibody leads to robust priming of CTL and anti-tumour immunity induction. Antibody-antigen-adjuvant conjugates targeted to DEC205 were superior in inducing antigen-specific killing compared to untargeted OVA-CpG conjugates. However, in the prophylactic and the therapeutic B16 pseudo-metastasis model, the antibody-containing conjugates were only slightly more efficient in preventing tumour growth. It will be interesting to evaluate whether the difference between the conjugates depends on the residual targeting specificity of the antibody-antigen-adjuvant conjugates.

## Discussion

The aim of this study was to generate conjugates for the targeted co-delivery of antigen and adjuvant to DEC205^+^ DC for the induction of anti-tumour immune responses. However, when comparing conjugates generated with anti-DEC205 specific antibody to equivalent conjugates containing isotype control antibody, it became evident that the CpG ODN moiety mediates binding to DEC205^−^ cells, thus partially overriding antibody-mediated targeting of DC. Interestingly, DEC205-independent CpG-mediated delivery of the conjugates containing isotype control antibody led to the induction of strong CTL responses for conjugates generated with antigen-specific peptide biochemically linked to the antibody, whereas in the case of conjugates containing antigen fusion antibody the level of CTL killing induced was minimal and significantly lower than for equivalent conjugate containing DEC205-specific antigen fusion antibody. Our data suggest that SIINFEKL peptide-mediated uptake in combination with CpG-mediated uptake of the conjugates rather than CpG-mediated uptake alone overrides the DEC205-mediated targeting and drives very efficient CTL induction by isotype control containing conjugates. Since the observed peptide-mediated uptake of conjugates represents a peptide-specific artefact and was not detected for conjugates containing peptides other than SIINFEKL or full-length OVA as present in antigen fusion antibody (Figure 3 and data not shown), we did not further investigate this phenomenon. What our data clearly show is that genetic fusion of the antigen is favourable over biochemical linkage for the generation of well-defined antibody-antigen conjugates and in order to avoid peptide-mediated effects. The CpG-containing conjugates generated with antigen fusion antibody were overall more efficient in inducing CTL responses and anti-tumour immunity and did so at lower doses as compared to the conjugates generated with SIINFEKL peptide (data not shown).

In the absence of SIINFEKL peptide-mediated binding, CpG-mediated delivery of antibody-antigen-adjuvant conjugates does not efficiently promote the induction of strong CTL responses as evidenced by the antigen fusion isotype control antibody-CpG conjugates. Firstly, this result suggests that despite of CpG-mediated conjugate delivery, part of the conjugate is delivered in a DEC205-dependent manner to cross-priming DC. Secondly, it supports the notion that DEC205-mediated delivery of antigen and in our case also of the adjuvant promotes cross-priming.

It also should be noted that the actual cross-priming DC population may be a subfraction of the mouse CD8α^+^ DC subset. Furthermore, CD8α expression is not a sufficient marker for distinguishing the different DC subsets in lymph nodes and comparing the uptake of the different conjugates by lymph node CD8α^+^ DC may be misleading [33]. This would explain why DEC-Fus-CpG conjugate is more efficient in inducing a CTL response than iso-Fus-CpG despite the fact that both conjugates are equally well taken up by CD8α^+^ DC in the draining lymph node. Another possible explanation for this observation is that DEC205-mediated conjugate uptake destines the antigen to a different fate than CpG-mediated delivery with the former promoting cross-priming more efficiently than the latter. In this context it is interesting to note that mannose receptor-mediated uptake has been described to advance cross-presentation of antigen in DC [34], since it transports the antigen into an early endosomal compartment from which cross-presentation can occur [35]. Like mannose receptor, DEC205 is involved in antigen processing and may favour uptake into early endosomes [36] and promote cross-presentation [37]. In contrast CpG, which has been described to mediate uptake via macrophage scavenger receptor type A [38], [39], [40], may deliver the conjugates to an endosomal compartment that is specialised in generating peptides for presentation on MHC class II.

It will be interesting to identify whether antibody-antigen-adjuvant conjugates targeting DEC205 and equivalent untargeted antigen-adjuvant conjugates show differences in their pharmacokinetics and whether these differences are entirely due to the difference in size or are mediated completely or in part by the antibody. The DEC205-specific ovalbumin fusion antibody used in this study does not allow for FcR binding [18], [41]. Thus, FcR-mediated effects are unlikely to contribute to the observed difference in CTL induction between the antibody-antigen-adjuvant and the antigen-adjuvant conjugates.

An alternative strategy for CLR-targeted co-delivery of antigen and adjuvant to specific DC is the use of liposomes [42] or biodegradable nanoparticles [43], [44] coated with CLR-specific antibodies and encapsulated with the antigen and the adjuvant. The encapsulated adjuvant does not alter the targeting specificities of these vaccines and the co-delivery of antigen and adjuvant clearly has advantages over the use of soluble adjuvant as shown for the targeted nanoparticle-based vaccine [44]. However, it is currently unclear how well such CLR-targeted liposome- or nanoparticle-based vaccines compare to an antibody-antigen conjugate approach with regard to the efficacy of the vaccination. Only direct comparison of the different approaches will allow an assessment of which strategy ultimately leads to the most efficient anti-tumour immunity induction. Until this is clarified it remains unclear which strategy will prove most promising in a clinical setting. Currently, it can not be excluded that antibody-antigen-adjuvant conjugates represent a more efficient means for inducing anti-tumour immunity despite their partial lack in antibody-mediated targeting specificity.

A better understanding of the underlying mechanisms that lead to successful delivery of conjugates to the cross-priming DC subset is required to inform optimal conjugate design. From our study, we conclude that conjugation of the adjuvant to antigen-fusion antibody can offer advantages over using soluble adjuvant despite the partial loss in targeting specificity.

## Materials and Methods

### Ethics Statement

All animal experiments were undertaken in accordance with UK governmental regulations (Animal Scientific Procedures Act 1986) under the project licences PPL 70/6317 and 70/7131. Ethics approval for the study was granted by the Guy’s Ethical Review Process committee. To minimise suffering of animals in particular upon implantation of tumour cells we followed the UKCCCR guidelines for the Welfare of Animals in Experimental Neoplasia [45].

### Oligonucleotides and Peptides

The phosphorothioate ODN CpG 1668 (TCCATGACGTTCCTGATGCT) and GpC 1668 (TCCATGAGCTTCCTGATGCT) were purchased from either MWG Eurofins (Ebersberg, Germany) or Thermo Scientific (Loughborough, UK) with a thiol modification (S-CpG and S-GpC). Unmodified CpG 1668 (CpG) and phosphorothioate ODN polydT17-G12 (T_17_G_12_) were from MWG.

Peptides were synthesized by Cancer Research UK (London, UK) or Thermo Scientific. The MHC class I-restricted OVA-specific peptide SIINFEKL (OVA_257–264_) was synthesized in unmodified form or with an added cysteine and biotin group at the C terminus (SIINFEKL-C-biotin). Likewise, the 36 amino acid long peptide OVAlong containing the SIINFEKL epitope plus the MHC class II-restricted OVA epitope (OVA_323–339_) was obtained including a cysteine and a biotin group at the C-terminus.

### Generation of Antibody-antigen-adjuvant Conjugates

The antibodies were purified from the supernatant of NLDC-145 (anti-mouse DEC205-specific rat IgG2a antibody), Y13-238 (anti-human p21-specific rat IgG2a antibody) and SFR3-DR5 (anti-human HLA-DR5-specific rat IgG2b antibody) hybridoma cells, all of which were purchased from LGC standards (Teddington, UK). Antibody purification was performed using the Montage Antibody Purification kit from Millipore (Watford, UK). Antigen fusion antibody was isolated from the supernatant of transiently transfected 293 T cells as described [18]. 293 T cells were obtained from Sophia Karagiannis’ group at King’s College London [46].

Antibody and antigen fusion antibody was treated with sulfosuccinimidyl 4-[N-maleimidomethyl]cyclohexane-1 carboxylate (sulfo-SMCC from Thermo Scientific) for 30 min at room temperature and excess crosslinker was subsequently removed using Amicon Ultra-4 filter units with a 10 kD cut-off (Millipore). S-CpG or S-GpC ODN was added to the activated antibody at a molar ratio of 12.5∶1. After an incubation of 20 min, peptide containing a C-terminal cysteine and biotin group was added to the antibody, but not to antigen fusion antibody, at a molar ratio of 6.25∶1 and the solution was incubated for another 70 min at room temperature. The generated conjugates were concentrated using Amicon Ultra-4 filter units (10 kD cut-off), followed by gel-filtration using a Sephacryl S-200 column (GE Healthcare, London, UK). The optical density at a wavelength of 260 nm and 280 nm of the collected fractions was determined and fractions containing conjugate were pooled. Conjugate was concentrated using Amicon Ultra-4 filter units (10 kD cut-off) and sterile filtered (Ultrafree-MC, Millipore). Antigen-adjuvant conjugates were generated as described [24].

The conjugates were characterised by SDS gel electrophoresis on Tris-glycin gradient gels (4–20%; Invitrogen, Paisley, UK) in combination with staining in ethidium bromide solution for nucleic acid detection followed by staining in Coomassie blue solution for protein detection. The CpG content of the conjugates was determined photometrically at a wavelength of 260 nm using a mixture of antibody and soluble S-CpG/S-GpC ODN as standard curve. The content of biotinylated peptide was established using the Fluoreporter assay from Invitrogen. Furthermore, conjugates were tested for endotoxin contamination in a Limulus amoebocyte lysate assay (Lonza, Slough, UK). The endotoxin concentration was consistently below 10 U/mg protein.

### Mice

C57BL/6 mice were obtained from Harlan UK (Bicester, UK), B&K Universal Ltd (Hull, UK) or Charles River (Margate, UK). TLR9 KO mice were bred at the biological service unit at King’s College London. Experimental procedures were performed on 6–12 week old mice and animals were sex- and age-matched for individual experiments.

### Binding Assays

CD11c^+^ DC were enriched from collagenase D/DNase-digested (Roche, Weleyn Garden City, Hertfordshire, UK) splenocyte preparations by positive selection with MACS beads (Miltenyi, Bergisch Gladbach, Germany). CD11c-enriched DC were stained with DyLight 488-labelled or unlabelled DEC205-specific or rat IgG2a isotype control antibodies or conjugates (0.3 nM–120 nM) in the presence of fluorescently labelled CD11c- and CD8α-specific antibodies (BD Bioscience, Oxford, UK) for 30–60 minutes on ice. In case of unlabelled antibodies or biotinylated peptide-containing conjugates, cells were subsequently stained with PE-labelled streptavidin (BD Bioscience). In case of unlabelled OVA fusion antibodies and conjugates, samples were indirectly stained with polyclonal biotinylated rabbit anti-OVA antibody (Abcam, Cambridge, UK) followed by PE-labelled streptavidin. Samples were analysed on a FACSCanto II (BD Bioscience) and the percentage or the MFI of DyLight 488-positive DC subsets was determined.

For blocking experiments unlabelled DEC205-specific antibody (100 µg/ml), unmodified SIINFEKL peptide (15 µM) or polydT17-G12 ODN (5 µM) was added to the antibody staining mix 15 minutes prior to addition of conjugates.

To investigate the uptake of conjugates in vivo, mice were injected with 5–8 µg DyLight 488-labelled conjugates into the footpad. Popliteal lymph nodes were dissected 6 or 16 h after conjugate administration and single cell suspension were prepared by liberase/DNase digest. Cells were stained with antibodies specific for CD11c, CD8, CD4 and CD3 (all BD Bioscience) in combination with anti-DEC205 (Miltenyi, Surrey, UK) antibody. Uptake of conjugate was determined by flow cytometry using a FACScanto II (BD Bioscience).

### Immunisation Studies

Mice were injected with conjugates into the hind footpad. In some experiments mice were co-injected with 25 µg anti-CD40 antibody (BD Bioscience), 1.5–30 µg soluble S-CpG 1668 ODN, 10 µg soluble CpG or 200 µg anti-DEC205 antibody. Control mice were injected with PBS. On day 5 or 6 after immunisation, mice were injected intravenously with 10^7^ target cells consisting of a 1∶1 mixture of SIINFEKL peptide-pulsed splenocytes labelled with 0.35 µM carboxyfluorescein succinyl ester (CFSE) and unpulsed splenocytes labelled with 3.5 µm CFSE. To analyse the in vivo CTL assay, mice were culled the next day and splenocyte suspensions were prepared by Liberase/DNase (both Roche, Welwyn Garden City, UK) digestion. The frequencies of the target cells pulsed with 200 nM peptide or left unpulsed were determined by flow cytometry for individual mice on a FACSCanto II (BD Bioscience) and the percentage of antigen-specific killing was calculated using the following formula: (1-%CFSE_peptide_/%CFSE_no peptide_)x100.

For ex vivo restimulation of CTL, splenocytes from immunised mice investigated by in vivo CTL assay were cultured in the presence of 1 µM SIINFEKL peptide over night. The next day, cells were incubated for 3 h in the presence of 5 µM Brefeldin before being harvested and fixed in 4% paraformaldehyde. Cells were stained with anti-CD8 and anti-Thy1.2 (or alternatively anti-CD3) antibody followed by intracellular staining of IFNγ (all antibodies from BD Bioscience). The frequency of IFNγ-producing CD8 T cells was determined by flow cytometry.

### Tumor Model

C57BL/6 mice were immunised into the footpad with conjugates as described above. 30 days after prophylactic vaccination, 4–5×10^5^ B16 melanoma cells expressing an OVA-GFP fusion protein were injected intravenously [47]. The OVA-expressing melanoma cell line had been generated earlier from parental B16 cells obtained from the Cancer Research UK Cell Service Laboratory by transduction with a VSV-G pseudotyped retrovirus in combination with single-cell sorting of GFP-positive cells. For therapeutic vaccination, mice were inoculated with 7.5×10^5^ of these OVA-expressing B16 melanoma cells and 6 days later mice were treated by footpad injection with conjugates as described above. The number of lung nodules was determined 18 days post tumour challenge with up to 250 nodules being counted per lung. Photographs of the left upper lobe of lungs fixed in Fekete’s solution were taken with a Fujifilm S5 Pro camera in combination with a Tamron 18–250 mm lens.

### Statistics

Statistical analysis was performed using a two-tailed Student’s t-test if not otherwise indicated (Prism Program; Graphpad software, La Jolla, USA). In the graphs p values are indicated as follows: * p<0.05; ** p<0.01; *** p<0.0001; n.s. not significant (p>0.05).

## Supporting Information

Figure S1 **Characterisation of antibody-antigen-adjuvant conjugates.** 2 µg DEC-OVA-CpG conjugate before gel filtration or 2 µg antibody mixed with 1 µg soluble S-CpG as control were run on 4–20% gradient SDS gels under non-reducing conditions. Gels were sequentially stained with Coomassie Blue (A) and ethidium bromide solution (B) for visualisation of proteins and nucleic acids, respectively. (C) The ethidium bromide-stained gel was loaded with 27 µg of DEC-OVA-CpG conjugate after purification by gel filtration.
(TIF)

Figure S2 **Immunisation with antibody-antigen conjugates plus soluble adjuvant.** C57BL/6 mice were immunised with 8 µg DEC-OVA or iso1-OVA conjugate with or without 10 µg of soluble CpG 1668 ODN by footpad injection. Control mice received PBS injections. At day 6 after immunisation, target cells were injected intravenously. The following day, the in vivo CTL assay was analysed by flow cytometry. Data are pooled from 3 experiments. The average percentage of antigen-specific target cell killing for each group is shown as a bar.
(TIF)

Figure S3 **Titration of antibodies and conjugates for staining of DEC205+ DC.** CD11c-enriched splenocytes from C57BL/6 mice were stained with 3–120 nM unlabelled (A, D) or DyLight-488 labelled (B, C) conjugates. As control cells were stained with 0.3–120 nM DyLight-488 labelled DEC205-specific antibody (B). In case of unlabelled conjugates, staining was performed indirectly using PE-labelled streptavidin which binds to the biotinylated OVA and OVAlong peptide (A) or by sequential staining with biotinylated anti-OVA specific antibody followed by incubation with PE-labelled streptavidin (D). In addition, cells were stained with CD11c-, CD8α-specific antibodies to distinguish DC subsets. Binding of conjugate was analysed by flow cytometry gating on CD8α^+^ CD11c^+^ DC. Background staining of CD8α^+^ DC not incubated with conjugate is shown as solid grey histogram. Data are representative of two independent experiments.
(TIF)

Figure S4 **Blocking of antibody conjugate binding.** CD11c-enriched splenocytes from C57BL/6 mice were incubated with 30 nM of conjugates in the presence or absence of unlabelled DEC205 antibody (100 µg/ml), polydT17-G12 (5 µM) or SIINFEKL peptide (15 µM) for blocking of antibody-, nucleic acid- or peptide-mediated binding of conjugates, respectively. (A) Unlabelled peptide-containing conjugates were stained with PE-labelled streptavidin in combination with CD11c- and CD8α-specific antibodies to distinguish DC subsets. (B) DyLight-488 labelled conjugates generated with antigen fusion antibodies were used in combination with CD11c- and CD8α-specific antibodies. Binding of conjugate was analysed by flow cytometry gating on CD8α^+^ versus CD8α^−^ CD11c^+^ DC. Background staining of DC not incubated with conjugate is shown as solid grey histogram. Data are representative of two independent experiments.
(TIF)

Figure S5 **Characterisation of antigen fusion antibody-adjuvant conjugates.** 10 µg of untreated antigen fusion antibody (DEC-Fus) and purified antigen fusion antibody-adjuvant conjugates (DEC-Fus-CpG and iso-Fus-CpG) were run on 4–20% gradient SDS gels under non-reducing conditions. The gel was sequentially stained with Coomassie Blue (A) and ethidium bromide solution (B) for visualisation of proteins and nucleic acids, respectively.
(TIF)

Figure S6 **Induction of antigen-sepcific killing and anti-tumour immunity after vaccination with a low dose of DEC-Fus-CpG conjugate.** C57BL/6 mice were immunised into the footpad with 1 µg of DEC-Fus-CpG conjugate (7 pmol of antigen) while control mice were injected with PBS. (A) At day 5 after immunisation, in vivo CTL assays were performed by intravenous injection of target cells. Antigen-specific killing of target cells was analysed the following day. The depicted data are pooled from 4 independent experiments. (B) 30 days post immunisation, OVA-expressing B16 melanoma cells were injected intravenously and 18 days later, the number of lung nodules was determined. Data from two independent experiments are shown. Each symbol representing an individual mouse while the average antigen-specific killing (A) or number of lung nodules (B) for each group is depicted as a bar.
(TIF)

Figure S7 **Decrease in the size of tumour nodules after therapeutic intervention.** Mice were inoculated intravenously with OVA-expressing B16 melanoma cells and 6 days later mice were vaccinated with different doses (7–56 pmol) of DEC-Fus-CpG or OVA-CpG conjugate by footpad injection. 18 days after tumour cell injection lungs were harvested, fixed and photographs of the left upper lobe were taken. The photographs show the lobes from one representative experiment out of three.
(TIF)
