# Supplementary figures and images for: Antibody-Antigen-Adjuvant Conjugates Enable Co-Delivery of Antigen and Adjuvant to Dendritic Cells in Cis but Only Have Partial Targeting Specificity
Source: PLoS One. 2012 Jul 10;7(7):e40208. doi: 10.1371/journal.pone.0040208 (PMC3393736; doi:10.1371/journal.pone.0040208)

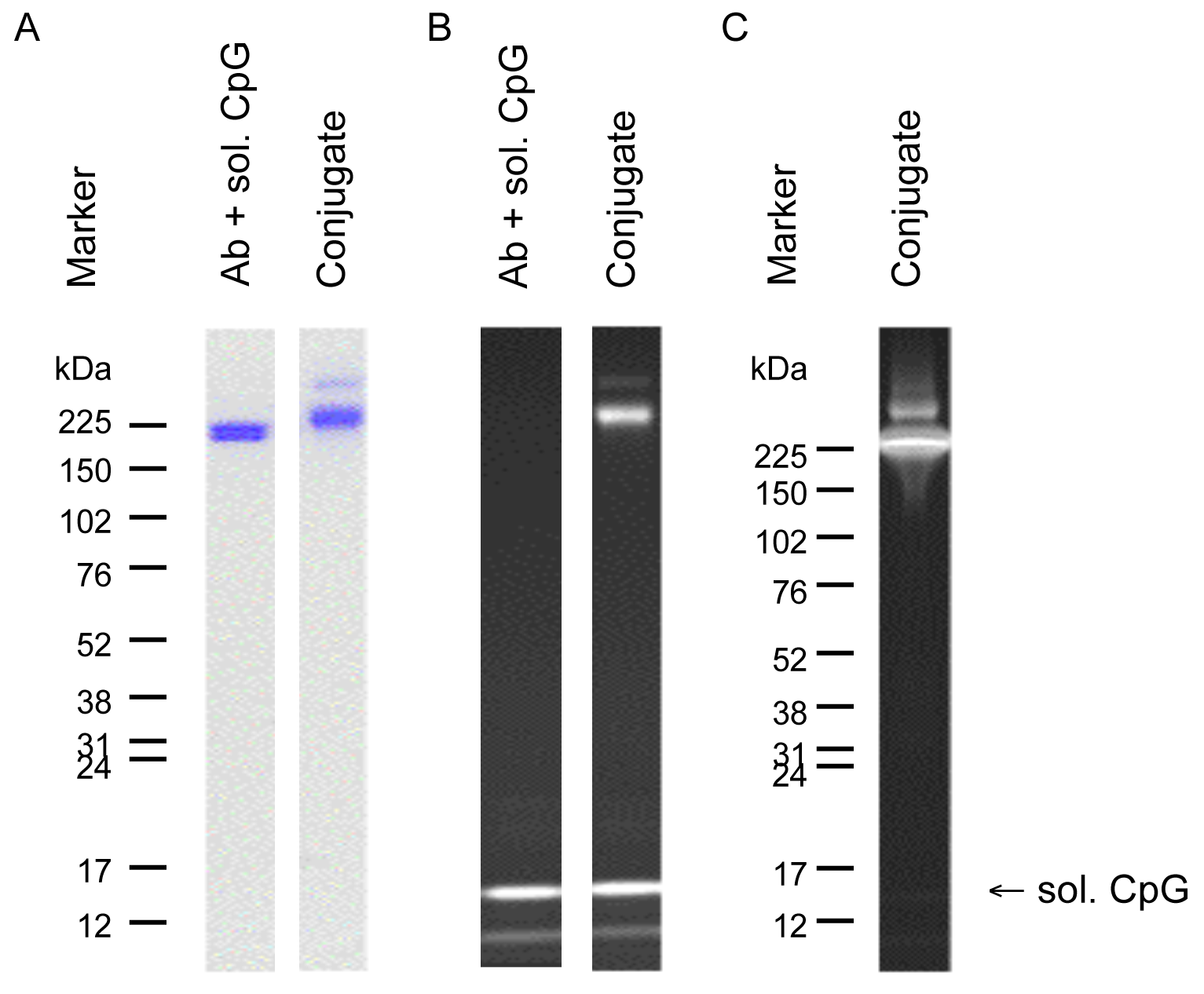

Supplement: Figure S1 — Characterisation of antibody-antigen-adjuvant conjugates. 2 µg DEC-OVA-CpG conjugate before gel filtration or 2 µg antibody mixed with 1 µg soluble S-CpG as control were run on 4–20% gradient SDS gels under non-reducing conditions. Gels were sequentially stained with Coomassie Blue (A) and ethidium bromide solution (B) for visualisation of proteins and nucleic acids, respectively. (C) The ethidium bromide-stained gel was loaded with 27 µg of DEC-OVA-CpG conjugate after purification by gel filtration. (TIF) [file pone.0040208.s001.tif]

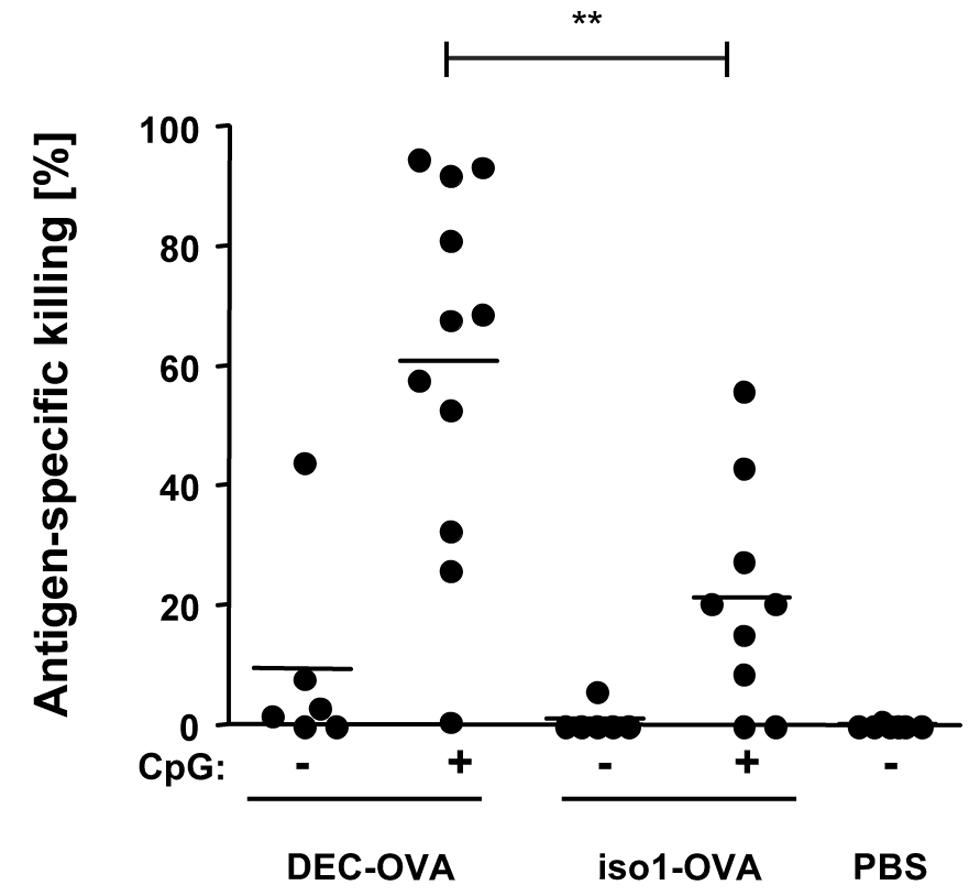

Supplement: Figure S2 — Immunisation with antibody-antigen conjugates plus soluble adjuvant. C57BL/6 mice were immunised with 8 µg DEC-OVA or iso1-OVA conjugate with or without 10 µg of soluble CpG 1668 ODN by footpad injection. Control mice received PBS injections. At day 6 after immunisation, target cells were injected intravenously. The following day, the in vivo CTL assay was analysed by flow cytometry. Data are pooled from 3 experiments. The average percentage of antigen-specific target cell killing for each group is shown as a bar. (TIF) [file pone.0040208.s002.tif]

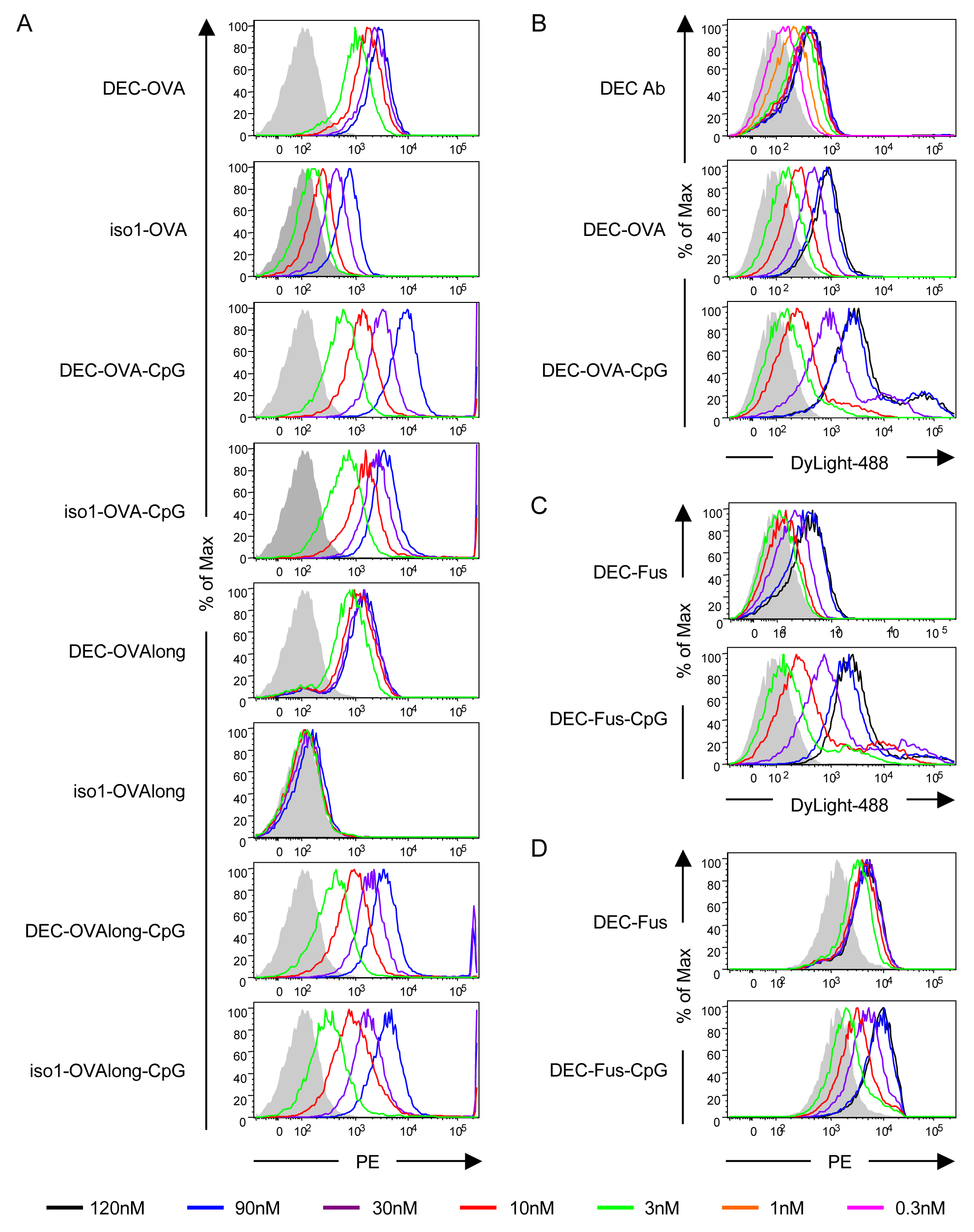

Supplement: Figure S3 — Titration of antibodies and conjugates for staining of DEC205+ DC. CD11c-enriched splenocytes from C57BL/6 mice were stained with 3–120 nM unlabelled (A, D) or DyLight-488 labelled (B, C) conjugates. As control cells were stained with 0.3–120 nM DyLight-488 labelled DEC205-specific antibody (B). In case of unlabelled conjugates, staining was performed indirectly using PE-labelled streptavidin which binds to the biotinylated OVA and OVAlong peptide (A) or by sequential staining with biotinylated anti-OVA specific antibody followed by incubation with PE-labelled streptavidin (D). In addition, cells were stained with CD11c-, CD8α-specific antibodies to distinguish DC subsets. Binding of conjugate was analysed by flow cytometry gating on CD8α+ CD11c+ DC. Background staining of CD8α+ DC not incubated with conjugate is shown as solid grey histogram. Data are representative of two independent experiments. (TIF) [file pone.0040208.s003.tif]

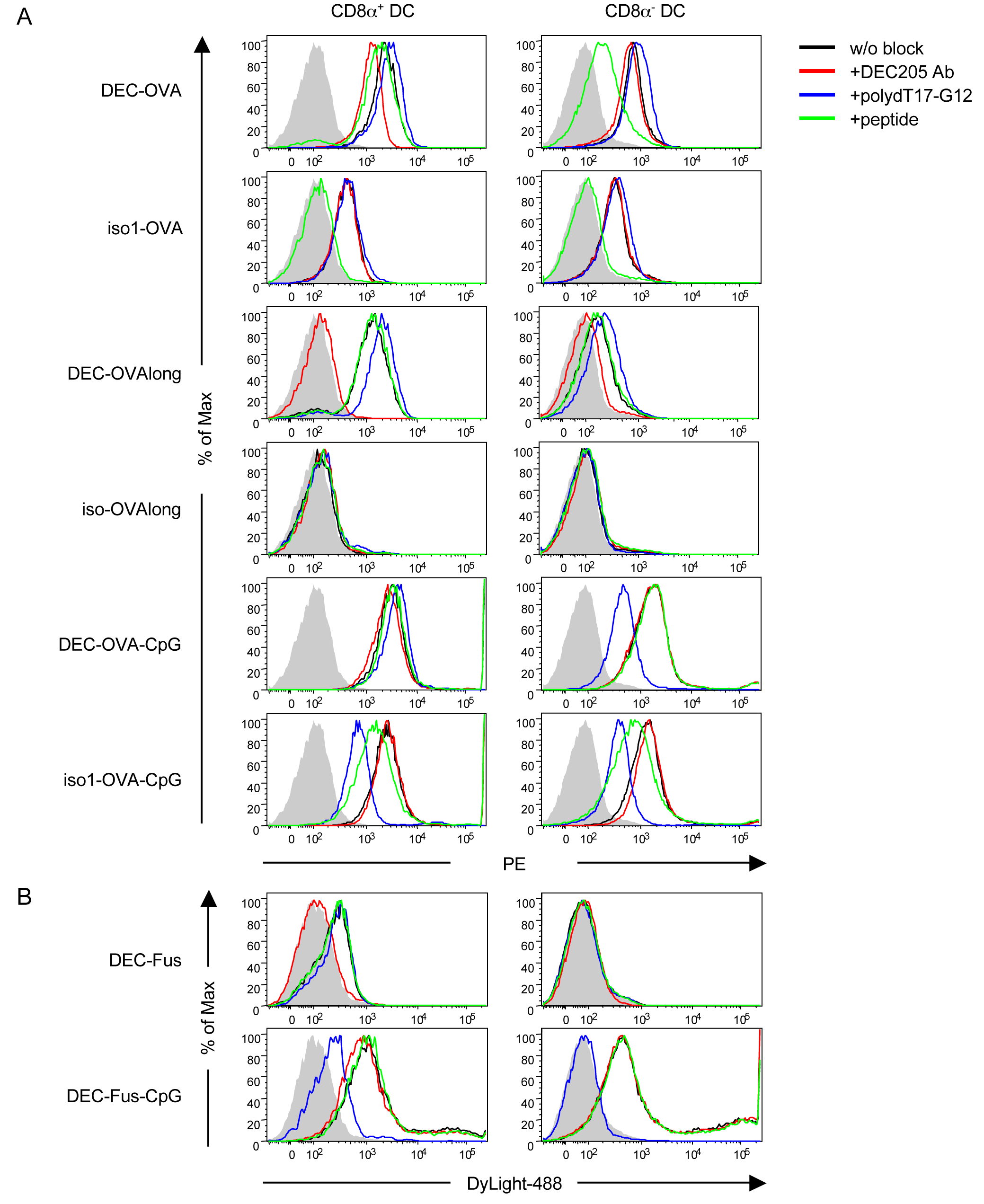

Supplement: Figure S4 — Blocking of antibody conjugate binding. CD11c-enriched splenocytes from C57BL/6 mice were incubated with 30 nM of conjugates in the presence or absence of unlabelled DEC205 antibody (100 µg/ml), polydT17-G12 (5 µM) or SIINFEKL peptide (15 µM) for blocking of antibody-, nucleic acid- or peptide-mediated binding of conjugates, respectively. (A) Unlabelled peptide-containing conjugates were stained with PE-labelled streptavidin in combination with CD11c- and CD8α-specific antibodies to distinguish DC subsets. (B) DyLight-488 labelled conjugates generated with antigen fusion antibodies were used in combination with CD11c- and CD8α-specific antibodies. Binding of conjugate was analysed by flow cytometry gating on CD8α+ versus CD8α− CD11c+ DC. Background staining of DC not incubated with conjugate is shown as solid grey histogram. Data are representative of two independent experiments. (TIF) [file pone.0040208.s004.tif]

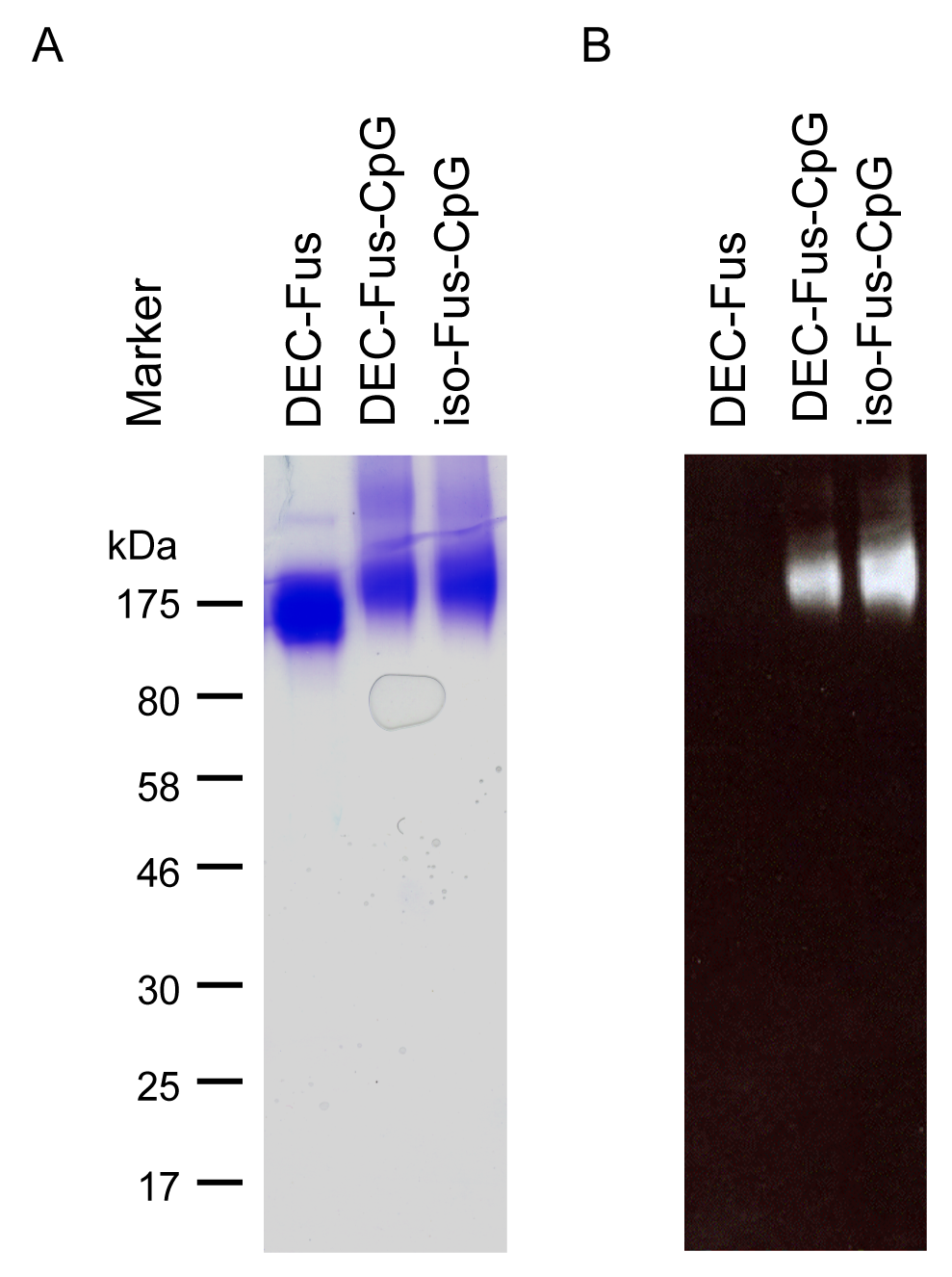

Supplement: Figure S5 — Characterisation of antigen fusion antibody-adjuvant conjugates. 10 µg of untreated antigen fusion antibody (DEC-Fus) and purified antigen fusion antibody-adjuvant conjugates (DEC-Fus-CpG and iso-Fus-CpG) were run on 4–20% gradient SDS gels under non-reducing conditions. The gel was sequentially stained with Coomassie Blue (A) and ethidium bromide solution (B) for visualisation of proteins and nucleic acids, respectively. (TIF) [file pone.0040208.s005.tif]

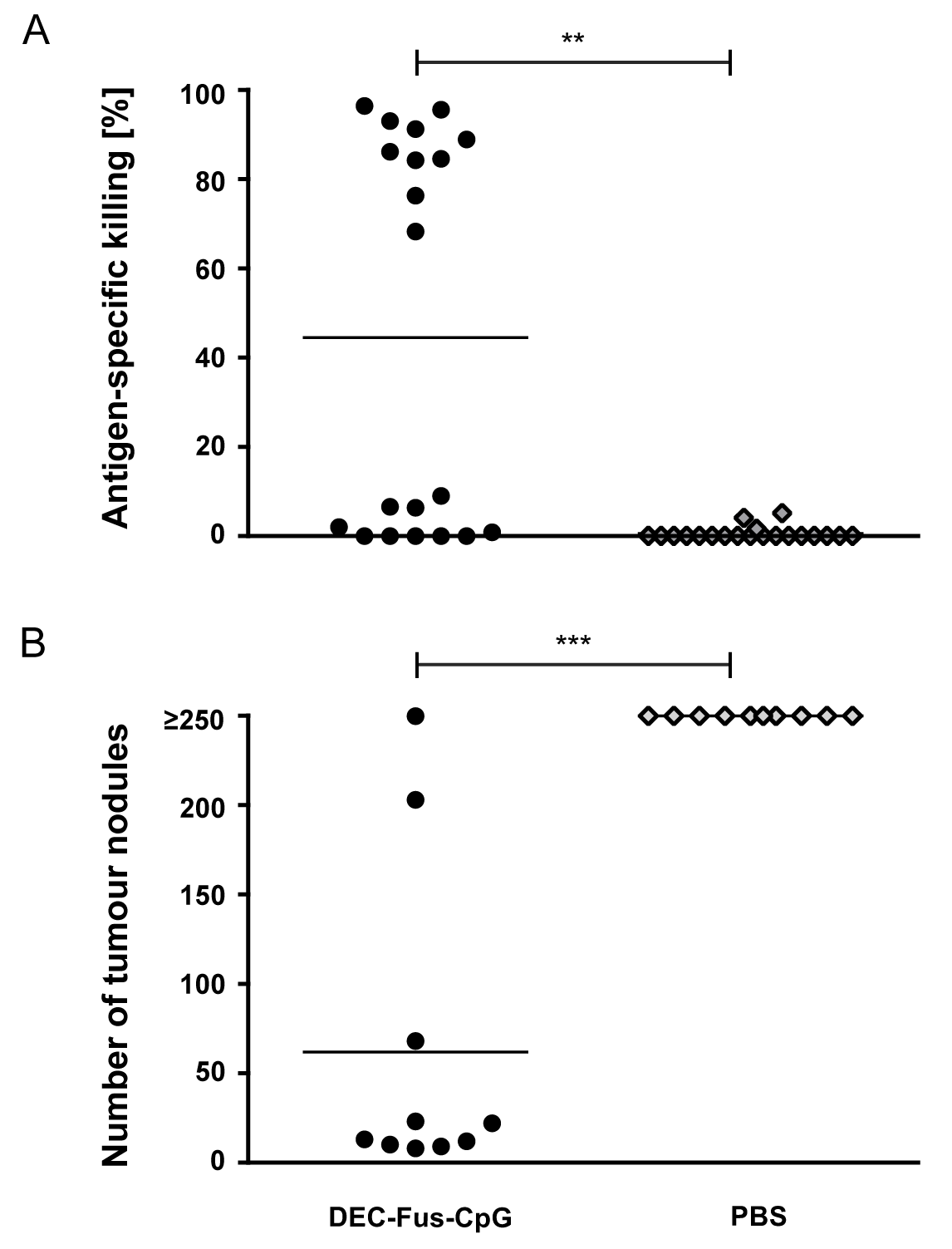

Supplement: Figure S6 — Induction of antigen-sepcific killing and anti-tumour immunity after vaccination with a low dose of DEC-Fus-CpG conjugate. C57BL/6 mice were immunised into the footpad with 1 µg of DEC-Fus-CpG conjugate (7 pmol of antigen) while control mice were injected with PBS. (A) At day 5 after immunisation, in vivo CTL assays were performed by intravenous injection of target cells. Antigen-specific killing of target cells was analysed the following day. The depicted data are pooled from 4 independent experiments. (B) 30 days post immunisation, OVA-expressing B16 melanoma cells were injected intravenously and 18 days later, the number of lung nodules was determined. Data from two independent experiments are shown. Each symbol representing an individual mouse while the average antigen-specific killing (A) or number of lung nodules (B) for each group is depicted as a bar. (TIF) [file pone.0040208.s006.tif]

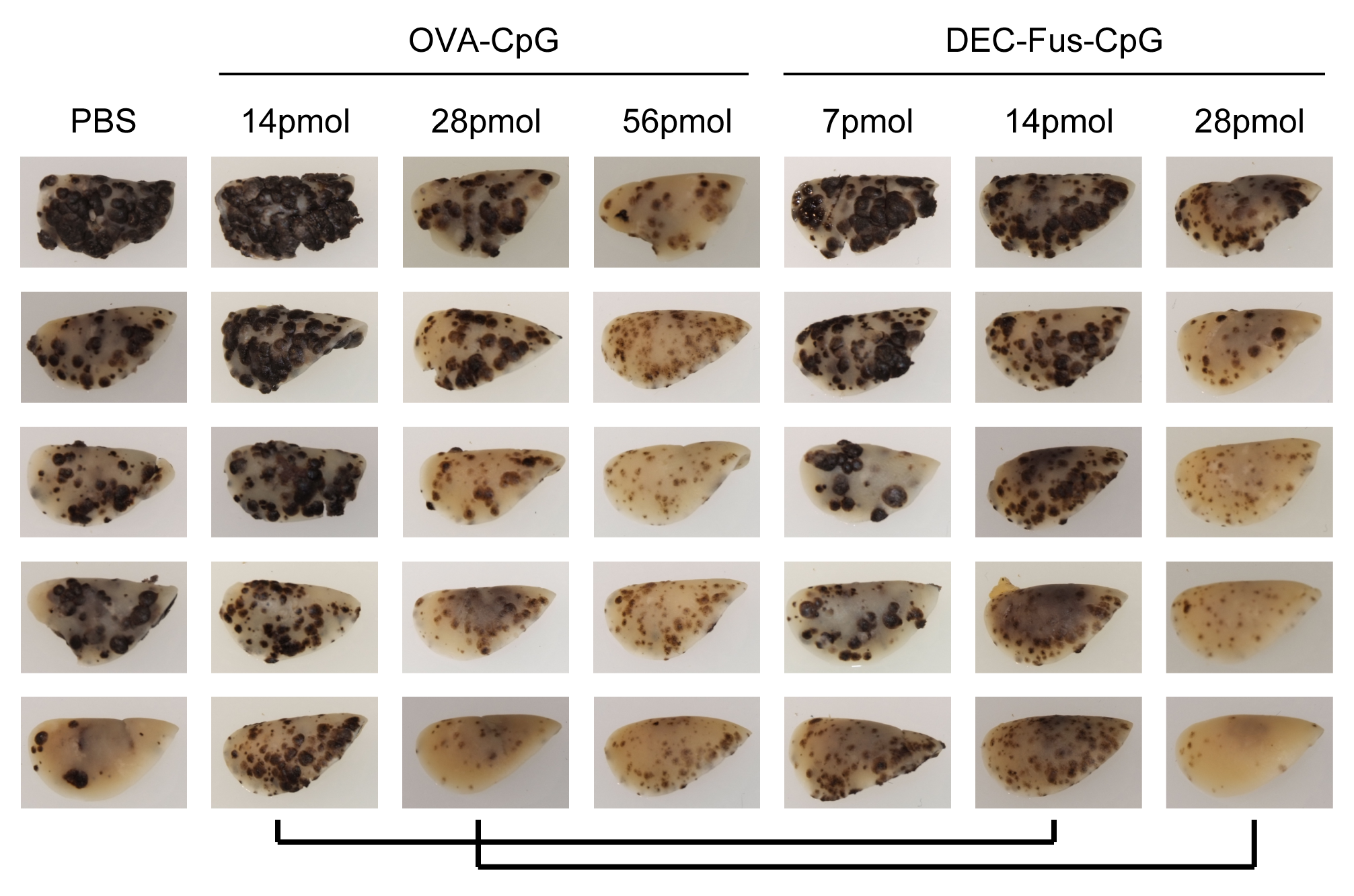

Supplement: Figure S7 — Decrease in the size of tumour nodules after therapeutic intervention. Mice were inoculated intravenously with OVA-expressing B16 melanoma cells and 6 days later mice were vaccinated with different doses (7–56 pmol) of DEC-Fus-CpG or OVA-CpG conjugate by footpad injection. 18 days after tumour cell injection lungs were harvested, fixed and photographs of the left upper lobe were taken. The photographs show the lobes from one representative experiment out of three. (TIF) [file pone.0040208.s007.tif]
